# Society Proceedings

**Published:** 1898-06

**Authors:** 


### SOCIETY PROCEEDINGS.

### PENNSYLVANIA STATE VETERINARY EXAMINING BOARD.

The regular June meeting for the examination of candidates for the license of the State Board to practise in Pennsylvania will be held at Philadelphia, in the City Hall, civil service examining board room, at 10 A.M. each day, June 20th and 21st.

By order of the President.

W. HORACE HOSKINS, President.

S. J. J. HARGER, Secretary, 205 N. Twentieth St., Phila.

### U. S. V. M. A.

DR. A. B. CAMPBELL, of Berlin, Ontario, expects to attend the U. S. V. M. A. meeting at Omaha. Canada should send a large delegation and its best representatives to this meeting to discuss the proposed change of name to American Veterinary Medical Association.

Among the more recent papers announced for the Omaha meeting are "Diseases of the Dog and Antitoxins," by H. D. Gill. V.S., New York College of Veterinary Surgeons; "A Study of the Healing Process in Ovariotomy in Cows," and "The Control of Hog Cholera," by Dr. M. H. Reynolds, of Minnesota State Board of Health; "Practical Points in Country Practice," by Dr. S. S. Whitbeck, and "Acute Indigestion in the Horse," by Dr. R. R. Bell, of the *American Veterinary Review*.

Clinical demonstrations have been promised of "Caudal Surgery," by Dr. H. D. Gill; "Extraction of Teeth and Other Dental Operations," by Dr. L. A. Merillat, of McKillip Veterinary College; "Ovarectomy in the Mare and Other Animals," by Dr. W. L. Williams, of the New York State Veterinary College; "Casting and Confining Animals," by Dr. T. S. Butler.

Transportation rates of one and one-third fare have been already obtained in the Western Passenger Association from Eastern terminal points of this district: Chicago, $20; St. Louis, $17; St. Paul, $15.75. Eastern associations are expected to afford favored rates.

Many Eastern veterinarians will take their wives with them to Omaha. Among the ladies there will be some new faces.

### CHICAGO VETERINARY SOCIETY.

MEETING called to order March 10, 1898, by the President, Dr. Walker. Roll-call showed fifteen members present. On request of Dr. Walker, Dr. Merillat took the chair as acting chairman. The minutes of the previous meeting were read by the Secretary. Exception to the same was taken by Dr. Walker relative to the last motion—an appeal from the chair. Motion by Dr. Campbell, seconded by Dr. Ryan, that the objectionable clause in the minutes of the previous meeting be excluded from such minutes; carried.

No report from the Secretary. The Treasurer reported $8.08 in the treasury.

The committee appointed to draft resolutions of regret on Dr. James Henderson's resignation reported through their chairman the resolutions adopted. After the reading of the same, it was moved by Dr. Merillat, seconded by Dr. Rishel, to accept same; voted and carried. Moved and seconded, that Dr. James Henderson be elected to honorary membership in the Society; carried. Motion by Dr. Nelson, seconded by Dr. Ryan, that the rules be suspended and the reconsideration of the previous vote relative to the expulsion or retention of Drs. Pierce and McGraw be acted upon; voted and carried. After some discussion it was moved and seconded to lay the reconsideration of the matter on the table; voted and carried. The second reading of the resolutions to change the quorum of the Society for the transaction of business from eleven to seven was read by the Secretary; voted upon and carried.

Under communications a letter was read from Dr. Albert Babb, Secretary of the Illinois State Veterinary Medical Association. Moved and seconded to notify Dr. Babb of the receipt of said letter; voted and carried.

Regular programme: Dr. Ryan, "Region of the Eye as Regards Examination for Soundness." The paper, although short, was a very good one, and great interest was shown by the members during its reading. Owing to the members' agreement with the essayist that all the conditions were unsoundnesses, no special discussion took place. Moved by Dr. Hughes, seconded by Dr. Nelson, to close the discussion; voted and carried.

Under new business, the resignations of Dr. E. L. Quitman and Dr. Frank Allen were presented to the Society by the Secretary. Motion by Dr. Dubia, seconded by Dr. Campbell, not to accept these resignations. Amendment by Dr. Hughes, seconded by Dr. Rishel, to defer action until the next regular meeting; voted and carried.

Moved and seconded to adjourn.

### *Dr. J. F. Ryan's Paper.*

THE EYES OF THE HORSE SUBJECTED TO CRITICISM IN EXAMINATIONS FOR SOUNDNESS. The eye, the organ of vision, the physiological mechanism of the sense of sight; the perfection of anatomical arrangement of parts by which optical images may be formed, or means by which the faculty of vision is exercised. Hence the necessity of being most particular in passing criticism on this most important organ.

A horse with impaired vision may become a very dangerous animal; he may see things imperfectly, distortedly, or not see them until just upon them (such as manhole covers in the streets, etc.), from which he unexpectedly shies, with very varying results. And again, on the other hand, a beautiful, clear, full, sound, intelligent eye enhances the appearance and value of the animal; and a common-place, dull, unsound, unintelligent eye detracts from his beauty and depreciates his value, from a lesser to very considerable extent as a consequence.

The following diseases and injuries to, and abnormal conditions encountered in the eye and its appendages I consider unsound:

*Opacities of the cornea*, of all forms, that are distinguishable on the corneal field.

*Cataract.* Any diminished transparency, or any opacity of the crystalline lens or its capsule.

*Palsy of Optic Nerve. Amaurosis* (amblyopia). From various causes; anæmia from illness or hemorrhage, lead poisoning, exposure to prolonged glare, as from snow-blindness, tumors, and other diseases of the brain implicating roots of optic nerve.

*Retinitis*, under pressure upon the retina from dropsical or inflammatory effusion, and also from overloaded stomach. Unsound except where disease is symptomatic of some removable cause.

*Periodic ophthalmia*, recurrent ophthalmia, constitutional ophthalmia moon-blindness—irido-choroiditis, in any stage of its periodicity is absolutely unsound.

*Loss of eye*, through injury, enucleation, rupture, etc.

*Absence of eyelid;* torn off, or removed in whole or in part.

*Absence of membrana nictitans.*

*Fungoid growths*, of whatever character, requiring excision.

*Occluded duct*, producing epiphora, requires surgical treatment, and is an unsoundness.

*Paralysis of lids* (ptosis), partial or complete, unsound.

*Strabismus* of all varieties, causing faulty position of the eye, requiring division of the tendon of the contracting muscle.

*Entropium*, or inversion of the eyelid.

*Ectropium*, or eversion of the eyelid. Both conditions require delicate surgical interference, and are unsound.

*Vermicular Ophthalmia*, caused by filaria papillosa, requires removal, and is an unsoundness.

THE meeting of April 14, 1898, was called to order by the President, Dr. Walker. On count only eight members were present. Three visitors were in attendance.

The minutes of the previous meeting were read and approved. The application of Dr. F. Lockwood Wingate for membership was duly approved and the doctor elected to membership.

Dr. Frank Allen read a paper on "Dental Cysts, Deafness, Paralysis of the Ear, Tumors on Cartilage of the Ear, Fistula, and Ménière's Disease." The paper was well received and called forth quite a discussion. On motion, the discussion was closed.

The resignation of Dr. E. L. Quitman was again laid over until next meeting.

The resignation of Dr. Frank Allen was presented for action. Upon request of the President, Dr. Allen withdrew the resignation.

Motion by Dr. Baker, seconded by Dr. Robertson, that the President hire the society room of St. Andrew's Society for the use of the Chicago Veterinary Society, so that we be enabled to have our own society room. Voted; carried.

On motion, adjournment.

*Dr. Frank Allen's Paper.*

DENTAL CYSTS. These may be found in all parts of the body, but generally in the sinuses of the head and near the base of the ear. They consist of a membranous sac containing developed or partially developed teeth. They would, in my opinion, constitute an unsoundness necessitating a surgical operation for their removal, although many animals pass through life without one and are still serviceable.

Deafness. This, from whatever cause, whether congenital or accidental, is a decided unsoundness. The causes of deafness in a horse are numerous, and not well understood. Since the overhead trolley wires have been in existence I have come across several cases from severe electrical shocks.

Paralysis. This, although a great disfigurement, I should hardly call an unsoundness, as a horse with paralysis of either one or both ears is just as good for service as a horse without. I should consider it a bad blemish, but not unsound.

Cartilage Tumors (Enchondromata). These tumors, if they should grow from the cartilage of the ear, although I have never seen one, I should consider a decided unsoundness, judging from my experience with them on the sternum, where, I believe, unless operated upon at a very early stage, they are incurable, and they seem to grow again faster than ever, after being removed.

Fistula. This is an unsoundness, in my opinion, necessitating a surgical operation. I have come across two of them recently, both passing from the inside of the conchial cartilage, running downward and forward about four or five inches. They both healed up nicely after being curetted out. I believe a common cause of these to be the use of a twitch on the ear.

Ménière's Disease (Labyrinth Vertigo). This disease I believe to be a most important one from a medical and legal point of view in an examination for unsoundness, for so far as I am aware there are no symptoms by which it may be detected unless the animal should be seized with vertigo while being examined, which would be improbable. That this disease occurs in our patients very frequently, I firmly believe, and also that many of us make an error in diagnosis by calling it megrims or staggers, and account for it by a reflex action from the intestinal tract; but surely if errors in diet were always the cause, megrims would be far more common with us than it is. What brought this disease prominently to my notice was the fact that I unwittingly purchased for my own use an animal affected with it, and which afterward showed all the symptoms of this disease as manifested in the human being. If you will pardon me for a few moments, I will give you the symptoms of this particular case, as I had an opportunity to hold a post-mortem on it, for the animal kindly committed suicide while in my possession.

After I had purchased the animal I asked the owner if he had any bad habits. He said no, except that he had "a bee in his bonnet." I drove him for awhile, and one day while climbing a hill at a walk he suddenly jumped to the right side and shook his head as if a wasp were in his left ear; staggered and would have fallen if I had not jumped to his head. When I went to put my hand up toward his ear it seemed as if his vision was distorted, for he shrank away as if he expected to get hit. The conjunctivæ were highly injected. In about fifteen minutes he was able to stand without help, and in half an hour fit to be driven. I tried dieting, bleeding, physic, bringing no results. On post-mortem, I found the Eustachian tube on the left side greatly reduced in size, as was also the guttural pouch and the membranous labyrinth in a state of inflammation, and about one-half the diameter of the right ear.

I will try to explain my theory for this disease:

I believe that it is caused by a hyperæmia of the membranous labyrinth and a partial occlusion of the Eustachian tube, and not a hemorrhage into the labyrinth, as suggested by Fleming, for if that were the cause, why should the symptoms be only transitory? the hyperæmia depending on, either, first a venous stasis from mechanical or other obstruction to the return current, or, second, an increased arterial supply. First, the causes of the venous stasis might be mechanical obstructions to the great vessels of the neck by collar, etc., the sudden lowering of the head, the venous current being retarded by gravitation; coughing, by increasing the thoracic pressure and so obstructing the passing of blood into the right auricle. Second, increased arterial supply produced by sudden physical exertion, rigidity of the walls of the arteries, by diminishing the elasticity and so increasing the pressure. The increase in the labyrinth blood-supply in the human is characterized by vertigo, impairment of vision, etc., and why not in our patients? We are all aware that the semicircular canals are closely connected with coördination of movement and equilibrium, as experiments prove that section of one side of them caused incoördination on that side, and section of both loss of equilibrium. Epidemic parotiditis is particularly apt to affect the labyrinth structures, and recent investigations prove this to be due to infection from the blood-current. The same disease would also affect the middle ear by partial occlusion of the Eustachian tube, thus rendering the air-pressure on the tympanum unequal; and knowing that this in a human being will give rise to symptoms analogous to labyrinth vertigo, why should it not do so in a horse? and I believe future post-mortems will prove that partial occlusion of the Eustachian tube is a frequent cause of this trouble. Of course, if this disease could be detected during examination, it would, in my opinion, constitute an unsoundness.

*Discussion.*

Dr. Walker: I owned a horse which, after my having him for about six weeks, tried to get his head through the wall of his stall one night. I got him out and sent him to pasture for about two or three months, and afterward sold him. Two or three weeks later he was hurt again, and I found him to be in the exact condition as at the time when I bought him. I think that there are some cases where it is quite possible to detect them. You will find them to be very bad leaders if you lead them from behind, and if a man tries to lead him with a halter he is inclined to pull back, but there are many cases where they deceive a veterinary surgeon.

Dr. Robertson: I have never been able to diagnose a case.

Dr. Baker: Mr. Chairman, I never had an opportunity of making an autopsy on a case of this kind, but they are comparatively not rare in practice. I rather admire Dr. Allen's theory with regard to the cause, especially after he had found an occlusion of the Eustachian tube. I think I could suggest as a possible additional cause of the hyperæmia of the labyrinth, that it might come directly from that, for the function of the Eustachian tube is to relieve the drum from excessive tension, and on this account there might be a great deal of tension of it. This naturally, especially in cases of violent sounds, would produce such violent movements of the drum as to increase pressure on the internal ear, and through that, presumably, a congestion takes place, so that with the fact existing, as proven by the postmortem, there is occlusion of the Eustachian tube in such a case, the result seems to be quite philosophical. In the paper, which I think, although short, was very good indeed, Dr. Allen speaks of congenital or accidental cysts. I could hardly imagine a cyst like that being accidental. I supposed they were always congenital. Then, regarding his conclusion regarding soundness or unsoundness in paralysis of the ear, if he recognized it as paralysis it is a diseased condition. I am inclined to call it an unsoundness, and I would not call a horse that has paralysis of one or both ears sound.

Dr. Allen: I think Dr. Baker makes a mistake in saying that I said that dental cysts are congenital or accidental. I said that deafness was congenital or accidental.

Dr. Baker: Speaking of congenital deafness, many cases of congenital deafness in both horses and dogs, which I found myself, were without any external opening. The skin was grown right over the auditory canal. Hearing was restored by simply cutting an opening through and removing the skin that occluded the external auditory canal. Dr. Allen did not refer to any particular cases of deafness either congenital or accidental.

Dr. Campbell: I would like to ask Dr. Allen just what condition we would find a horse in paralysis of the ear. What is it, a lop ear?

Dr. Allen: What I have seen in cases of paralysis of the ear has been in conjunction with paralysis of the lip, and there was always a drooping of the ears, which I would call a lopping.

Dr. Campbell: Did you ever hear of horses having had something put in their ears that were in that condition?

Dr. Allen: I have seen cases of that kind, but they would recover after removal of the foreign substance.

Dr. Wingate: I have seen cases in the West Indies where it is called lop-ear. I do not know whether it is due to paralysis. It is due to a very large tick that is found there, and if not looked to in time is apt to destroy the ear, and sometimes the ear drops off. This tick seems to be very fond of the ear, and it is very prevalent out there. In fact, they are so prevalent that nearly every time before grooming the horse they look after the ticks first and remove them.

Dr. Walker: I cannot agree with Dr. Allen that ears with paralysis are sound, though I have seen many lop-eared horses that I consider sound. I remember one that I saw some two years ago. He was taken into a blacksmith-shop, and he was somewhat ugly to shoe. They put a twitch on him, but it slipped off. The blacksmith then hit him with it, and he had paralysis of the ear. I saw him several hours later and pronounced him to be an unsound horse. There are several breeds of horses lop-eared; for instances, the mule, or the jack, but I think when it comes down to a horse with paralysis of the ear, I consider him an unsound animal.

Dr. Campbell: Mr. President, this Ménière disease I never heard of until Dr. Clancy gave me the list, and I would like to ask Dr. Allen if there is much of it.

Dr. Allen: I think that nine out of every ten cases of staggers are Ménière's disease. You will find it mentioned in two or three books on the human being. When I had this horse some two years ago, there was quite a discussion in England over this disease. Dr. Fleming read a paper on it before the Central Veterinary Medical Association in 1885. He says he never made a postmortem examination. None of the gentlemen present had ever done so.

Dr. Robertson: Mr. Chairman, I am inclined to favor Dr. Allen's philosophy on this disease, especially in regard to these so-called cases of staggers. In all cases that I have run across, I find it to differ from staggers. I have seen different digestive troubles, such as partial loss of sight, also pressing the head against the wall, etc., following indigestion or some trouble of that kind; but I have never seen this peculiar shaking of the head that would indicate an ear-trouble. Horses that I have known that were attacked in this way have been always properly taken care of, and their previous history was all right. Their digestion was good. I do not see how we get this peculiar shaking of the head unless from ear-trouble. I think the Doctor's philosophy is correct, and it would be well for us to investigate future similar cases.

Dr. Allen : In one of my cases the only treatment of any effect was hypodermic injections of pilocarpine, and this helped only temporarily. I tried bleeding. Physicked him; in fact, tried everything without any effect. Hot or cold weather made no difference.

THE meeting, May 12, 1898, was called to order at 8 P.M., by President Walker. Thirteen members were present. The minutes of the previous meeting were read and approved. No report from the Secretary.

Treasurer's report showed $16.43 in the treasury. The Secretary was requested to read the list of delinquents; they numbered twenty-nine, some of whom, however, had left the city.

Motion by Dr. Johnson, seconded by Dr. Robertson, that the Secretary notify the members two years in arrears that unless their accounts are paid by the next meeting they shall be dropped from membership; carried.

Dr. E. L. Quitman desired to withdraw his resignation. On vote he was allowed to do so.

The regular programme in regard to "Soundness of Horses" was led by Dr. Hughes. The following subjects were discussed: Splints, sprained tendons, sprained suspensory and check ligaments, diffused periostitis, osselets, neurectomy, sprung-knees, interfering, and firing. The subjects were thoroughly discussed. On motion, the discussion was closed.

Motion by Dr. Hughes, seconded by Dr. Ryan, that no meetings be held during July, August, and September; carried.

Motion by Dr. Campbell, seconded by Dr. Allen, that the President call upon the officers of the St. Andrew Society and ascertain what rental by the year they will accept for the room we now occupy; carried.

Under new business a letter was read by the Secretary from our former member, Dr. James Henderson, now of Scotland, setting forth his thanks for the resolutions of this Society in his behalf, and wishing the Society all prosperity.

A letter was also read from the Civil Service Commissioners of Chicago, stating they did not know when an examination for police veterinarian would be held.

On motion, adjournment.

*Dr. Joseph Hughes's Paper.*

SPLINTS. The great scope of the subjects assigned to me causes me to deal with them extemporaneously rather than prepare a paper on such a comprehensive number of conditions. To begin with, I have some rather positive opinions regarding splints and their relation to soundness and unsoundness. At a certain stage of its growth a splint is merely a localized periostitis attended with a limited amount of effusion. Later on a bony exostosis appears on the site of the periostitis. During the stage of effusion lameness is present. As soon as the exostosis appears, lameness disappears. A formed splint, then—that is, a matured, a developed splint, a splint that is clearly apparent and could be detected by any novice, rarely causes lameness. I say rarely, because I recognize the fact that we sometimes find a splint of very considerable extent propped up against the lower row of bones of the knee, which may interfere with the action of the joint or set up a localized synovitis. This is an instance in which a formed or mature splint produces lameness. When, however, a splint is placed below the line of the carpal articulation and having no relation with it, no matter whether it involves the lateral aspect of the metacarpal bone, the furrow between the large and small metacarpal bones, or the lateral aspect of the small metacarpal bone, I make the statement that it is harmless and will not give rise to lameness. Even in those cases involving the posterior border of the small metacarpal bone and slightly bending over the tendon, in my opinion, they rarely interfere with the usefulness of the animal, except during the stage of their development. When I examine a horse for soundness, and a splint of considerable dimensions is present on the inner aspect of the metacarpal region, say midway between the fetlock and knee, the purchaser generally puts the question, "Will the horse get lame from that splint?" If I find that the horse's action is clean and there is no tendency to interfere with the growth during motion, I unhesitatingly say, no. Sometimes splint-lameness afterwards develops in that particular limb, but when a careful examination is made the developing splint will be usually found to affect the *outside* of the region of the canon-bone rather than the inside. In my opinion, horses should be rejected that have developed splints close up to the knee, but we can safely overlook those exostoses when further down. There are many other points connected with the location of the splints and their relation to soundness, such, for instance, as splints located in the channel between the small and large metacarpal bones, the consideration of which, I hope, will be brought out during the discussion which will follow.

THICKENINGS OR SPRAINS OF TENDONS AND SUSPENSORY AND CHECK-LIGAMENTS. It may be stated broadly that these conditions give rise to unsoundness. A thickened tendon or ligament means a shortened tendon or ligament, owing to organized effusion in the interfibrous structure; and it is plain that a horse with a shortened tendon or ligament, cannot be as useful as one in the normal condition. In deciding as to whether a thickening involves the fibres of the tendon, or the peratendineum, one must depend upon the amount of flexion present in the limb while in the quiescent state; upon the action of the animal during movement, and upon digital manipulation of the part. Thickness in this region should be always looked upon with suspicion. Remarks made would apply to the condition known as bowed tendon. In racehorse practice, where sprains of tendons and ligaments are common and horses are continuously changing hands, we are often called upon to give an opinion on the seriousness of thicknesses affecting these tendons and ligaments. Thicknesses, however slight or insignificant, unless they are confined to the paratendineum, are very serious in these animals. In my opinion, strain and thickening of the inferior carpal or check-ligament is the most serious of all. Next to this is thickening of the suspensory, then thickening of the perforans, and lastly of the perforatus.

"BUCKSHINS" OR "BUCKED SHINS," is a condition that one might say, is entirely confined to racehorses, and, being a diffused periostitis, we often find exostosis involving the lower and anterior extremity of the metacarpal bone. Lameness is, as a rule, only present in the acute stage. Sometimes, however, the exostoses encroaching upon and involving the synovial membrane of the fetlock-joint produce more or less permanent lameness. These lower exostoses are known in racing parlance as osselets. The racehorse affected with them should be considered unsound, more especially if the animal is under four years old; in aged horses, where there is no lameness associated with their presence, I think they may be overlooked.

SPRUNG-KNEES, on account of limiting the animal's usefulness, and on account of detracting markedly from the appearance of the animal, beside having a tendency to cause stumbling, should be regarded as unsound. I am aware of the fact that there are horses with badly sprung knees which give the utmost satisfaction in the work which they are required to do. Horses having an oblique shoulder-blade with sprung knees do not usually stumble, and in passing on the seriousness of the condition the conformation of the shoulder should always be considered. I hold, however, that the usefulness of an animal having sprung knees is markedly interfered with, and the condition is often associated with disease of other parts, and, as such animals frequently stumble, they should be rejected.

SCARS OF NEUROTOMY. In examining the region of the canon it is unnecessary for me to say that scars resulting from an operation of neurectomy are sufficient cause to reject the animal.

FIRING. The mark of a firing-iron is not neecssarily an evidence of unsoundness. For instance, an animal may have been fired for what was supposed to be splint-lameness, when perhaps the lameness might not have been located at that point, or the animal might have been fired for a supposed tendon-trouble. Should we find, on examining such an animal, that there is no lesion of tendon, ligamentous, or osseous structures, and that the prospective purchaser does not object to the scars made by the iron, we should consider the region sound.

INTERFERING between the knee and fetlock should be regarded as unsoundness. Of course, in arriving at such a conclusion we should take into consideration the general conformation of the forelimbs. Animals which toe out and stand base narrow are most prone to this trouble The condition is still more aggravated if the chest is narrow. Horses with such conformation should be rejected in every instance.

*Discussion.*

Dr. Hawley: I wish to make a remark on one point in regard to interfering. I think I can state positively that about eight out of ten horses that come from the country to the city, and have never been shod behind, will interfere with the first pair of shoes. If you were to condemn all horses that interfere you would have to condemn eight out of ten of these cases.

Dr. Hughes: Interfering as I dealt with it covers interfering between the fetlock and knee only—hitting the shin.

Dr. Walker: Regarding neurectomized horses, you say you would in all instances reject them.

Dr. Hughes: Yes, in every instance.

Dr. Walker: I was consulted a few days ago on a case like that. A party called at my office requesting me to examine a horse for him, belonging to a doctor, that he was going to purchase. The doctor asked two hundred dollars for him. I saw that the horse was neurectomized. The doctor admitted that his horse went lame some time ago, and that his veterinarian stated that there was a nodule on the nerve which had to be taken off. After this was done the horse went all right. I advised the party not to buy the horse.

Dr. Robertson: In regard to the question of shaky knees. Now, I have known many very good horses kept in a barn, especially in box-stalls, during the winter, when suddenly put to work in the spring develop shaking of the knees. I had several cases this summer; one in particular had it in a very marked degree. She was blistered in her back tendon, and has since done her work well. Therefore, one would have to be careful that he should know something of the history of the case before he rejects a horse. Regarding interference, I would echo the sentiments of Dr. Hughes. Of course, interfering of the canon-bone is unsound. I agree with Dr. Hughes that up to within the last year or two horseshoers made a great mistake when shoeing green horses by lessening the circumference of the foot to such an extent as to cause interfering. I would also suggest that in examining a horse, one be very careful if a green horse has been shod and is afflicted with over-reaching. The trouble was that the horseshoer has cut a large amount of the wall, making it as low as possible and raised the horse's foot up behind and also the toe in front, and the consequence was that he clicked at every step he made. As soon as he changed this it lessened the interfering to a very perceptible degree. Use judgment in regard to the amount of wall that is to be taken off and the kind of shoe put on on a green horse. I would recommend for a green horse to cut off just as little of the wall as possible and shoe him as light as possible.

Dr. Ryan: Did you not find that in most cases of sprung-knees a great deal is due to the conformation? The conformation is not correct.

Dr. Hughes: Decidedly. Anyone on a breeding-farm will have noticed it in colts growing up right from colthood. It is a very common thing to find, and he may have a perfect conformation of the shoulder. Standing a horse in a stall with an inclined floor is a common cause. The opinion of some people is that he will straighten up again. I say that in every instance one of those should be condemned.

Dr. Hawley: Supposing one buying a horse would buy him for the show ring; he was an extra highgoing horse of beautiful appearance, slightly sprung in the knees; a horse that the judge in the show-ring would not condemn; a horse that would pass in the show ring as sound. Would he have to be condemned by the veterinarian?

Dr. Hughes: If I were the veterinarian I would condemn him, for if it comes to a question of downright soundness, you would have to throw him out.

Dr. Robertson: I was called a few weeks ago to treat a horse of splendid conformation and high action. He was standing all winter idle, and was remarkably shaky after the first drive. His knees just quivered. The owner told me he gave him a very hard drive. I recommended rest for a few days, and after treating him he seems to be doing all right. Would you have pronounced him unsound on account of this little shaking of the knees?

Dr. Hughes: My old preceptor gave me good advice in cases of this kind. That is, if you find an instance where you suppose the trouble or lesion is of recent occurrence, be fair to the dealer and to the buyer, and bring them together and say, "Now you can wait for this horse for a week or two. He is a little off, and may be all right in a short while."

Dr. Ryan: In regard to horses cut out in the knees, do you consider this as much a defect as over in the knee position, and do you consider them unsound?

Dr. Hughes: I do; but not as much a defect as going over at the knees. Everybody knows what going over at the knees is, while one cut out in the knees is very seldom noticed. An everyday horse could be possibly passed, but a fine horse I would reject.

Dr. Greiner: Do I understand right, that Dr. Hughes pronounces a horse with a splint without lameness as sound? None of the people that I have to deal with care to buy a horse with a splint.

Dr. Hughes: In answer to this, I would say that Dr. Greiner must educate his people to tolerate splints. I do not attach the slightest importance to the presence of developed splints when they are away from the knee.

Dr. Hawley: In regard to the question of unsoundness, especially small defects. Now, a man that deals in light-harness horses, for instance, is no more a thief than the one who buys them, and, therefore, should be given the same consideration. I claim that no man is fit to examine horses for soundness until he has gone on the market and purchased horses extensively; therefore, a veterinarian in examining horses for soundness ought to consider both sides of the question before he rejects a pair of horses.

L. Campbell, D.V.S.,
Secretary.

### NORTH CAROLINA VETERINARY MEDICAL ASSOCIATION.

Meeting was called to order by the President, C. R. Ellis, at Greensboro, December 27, 1897. A fair number of members responded to roll-call. Address by the President showed the great necessity of strict sanitary regulations in our State as to meat- and milk-inspection. Discussion of this paper led to a motion to appoint each member a committee to aid the Secretary in procuring the signatures of all physicians in the State who would aid in securing said regulations.

Committee on Legislation was appointed, consisting of the President, Secretary, and Dr. W. C. McMakin. Board of Examiners: Drs. Charles H. Lockwood, H. G. Bessent, and H. F. Baüer, one to serve three years, one two years, and the other one year.

Mrs. C. R. Ellis was elected an honorary member, on account of the interest shown by her in the Association.

Dr. John Lockwood, of Washington, D. C., was elected to membership.

The Association adjourned to meet in Wilmington in July.

J. W. Petty,
Secretary-Treasurer.

### VETERINARY MEDICAL ASSOCIATION OF NEW YORK COUNTY.

In the absence of the President and Vice-President, this Association was called to order May 4th by the Secretary, at 8.45 P.M. at the Academy of Medicine.

The Secretary then asked the members to appoint a chairman to conduct the meeting. It was then regularly moved and seconded that Dr. Gill act as chairman *pro tem.;* carried. Dr. Gill, thereupon, took the chair and proceeded with the regular order of business. The following members responded to roll-call: Drs. C. C. Cattanach, J. S. Cattanach, J. S. Cattanach, Jr., Delaney, Ellis, Gill, Grenside, Lamkin, Machan, MacKellar, and O'Shea. The minutes of the previous meeting were read and approved.

Dr. Gill, chairman of the Board of Censors, stated that as a quorum of that committee was not present they could not take action on important business in hand; therefore they had no report to offer to the meeting.

Dr. Gill reported for the Ways and Means Committee, in the absence of their chairman, that Dr. Lellman will read a paper at the June meeting on "Multiple Sclerosis of the Brain and Spinal Cord of the Dog," and a second essayist will be procured for the same meeting.

Dr. O'Shea, chairman of the Judiciary Committee, reported that the Governor had signed the jury bill and it is now a law. This report brought forth the applause of the members present.

Moved and seconded, that the reports of the various committees be accepted; carried.

Dr. J. S. Cattanach read a paper entitled "Economy in the Practice of Veterinary Medicine." A free discussion followed by all the members present.

Dr. Lamkin then read a paper on "Parturient Apoplexy." This paper also met with a free discussion.

Moved and seconded, that a vote of thanks be tendered to the essayists; carried.

Moved and seconded that the meeting adjourn; carried.

Robert W. Ellis, D. V. S.,
Secretary.

### KEYSTONE VETERINARY MEDICAL ASSOCIATION.

The May meeting was called to order by President Pearson at 8 P.M. on the 10th ult. The following members of the profession were present: Jas. D. Mecray, S. J. J. Harger, H. D. Hackler, Otto von Lang, Wm. J. McCoy, W. B. Stauffer, J. Price Vasey, John B. Raynor, Geo. Felton, M. W. Drake, C. J. Marshall, John W. Adams, J. D. Houldsworth, W. H. Ridge, Leonard Pearson, Walter L. Hart, Jas. B. Rayner, T. B. Rayner, A. N. Lushington, W. L. Rhoads, W. Horace Hoskins, C. T. Goentner, Chas. Williams, and Chas, Lintz; also, Dr. J. Cheston Morris.

After the general routine business Dr. W. Horace Hoskins gave a most interesting talk on "Emphysema," covering his subject so well that President Pearson could scarcely get a word from any of the members or visitors on the subject, there being very little ground even for questions, yet discussion brought out diverse opinions as to the question of lesions and treatment. Dr. Pearson then opened the subject of "Army Veterinary Service" by telling of the rank, promotion, services, and requisites of the army veterinarians abroad; he also proved that he had given this subject much study and thought, and went at it in his thorough, careful way, giving a complete history of the army veterinarian's position, rank, progress, and education in the Roman, Russian, German, and English armies from the time of Cæsar to the present day. He cited facts to show that the loss due to inadequate veterinary services during the time of war would pay for a well-equipped system three or four times. The discussion on this subject brought forth a number of views as to the best method of advancement.

Dr. W. Horace Hoskins moved that the officers of the Keystone Veterinary Medical Association confer with the officers of the State Association as to the best methods by which $200 might be raised from the profession throughout the State for the furtherance of army legislation on behalf of army veterinary rank. The President appointed Drs. Hoskins and Rhoads as a committee to take this in charge. Dr. Rhoads moved that the President appoint a committee of three to draft resolutions directed to Dr. Salmon, and pledge the support and assistance of the Keystone Veterinary Medical Association in this work. The President appointed Drs. Hoskins, Marshall, and Allen on this committee.

After the report of the case the application of Dr. Jas. D. Mecray, of Maple Shade, N. J., was considered; having been favorably acted upon by the Board of Censors, he was unanimously elected to membership.

Dr. Pearson appointed Dr. W. Horace Hoskins to report at the following meeting on current veterinary topics that would be of interest to the Association.

The Association then adjourned, to meet June 14th, at which time Dr. Harger will open a discussion on "Quittor," and Dr. Adams will open discussion on "Surgical Methods on Cribbing." W. L. RHOADS,

Secretary.

### MASSACHUSETTS VETERINARY ASSOCIATION.

THE regular monthly meeting was held at No. 19 Boylston Place, Boston, January 25, 1898, President Winchester in the chair. Members present: Drs. Beckett, Cronon, Cutting, Emerson, Frothingham, Hamilton, Lee, Lewis, McLaughlin, Parker, Pierce, Soule, Winchester.

Dr. McLaughlin reported for the Legislative Committee that he thought with the coöperation of each member of the Association the Veterinary Bill would become a law at this session.

The essayist for the evening was Mr. Daniel S. J. Murphy, a student in the Veterinary Department of the Harvard Medical School, who read a valuable paper on "Roaring." A general discussion followed.

Adjourned at 11.30 P.M.

THE fourteenth annual meeting was held April 27th at the Quincy House, Boston, at which there was a large attendance of members.

A business meeting was held in one of the parlors, and the following officers were elected for the ensuing year: President, John F. Winchester, D.V.S., of Lawrence; First Vice-President, E. H. Holden D.V.S., of Springfield; Second Vice-President, S. R. McLaughlin, D.V.S., of Newton; Secretary and Treasurer, Henry S. Lewis, M.D.V., of Chelsea. Executive Committee: Langdon Frothingham, M.D.V., of Boston; L. H. Howard,

D.V.S., of Boston; Howard P. Rogers, M.D.V., of Allston; George Lee, D.V.S., of Brighton; P. J. Cronnon, M.D.V., of Boston.

The Secretary's report showed seven new members during the last year.

The Treasurer reported the expense for the year just passed, $187.80; received for dues for the same period, $170.00; on deposit in bank, $274.86.

The members then adjourned to the banqueting hall with their guests, Mr. Leander Herrick, of the Massachusetts Cattle Commission, and Mr. D. S. J. Murphy, of Cork, Ireland.

The President introduced Dr. J. R. McLaughlin as toastmaster, and the following toasts were responded to: "Our Country's Arms—may they be successful," Dr. Austin Peters; "Our Alma Mater—Harvard University," Dr. F. H. Osgood; "American Veterinary College," Dr. L. H. Howard; "McGill University," Dr. John M. Parker; "Massachusetts Cattle Commission," Mr. L. Herrick; "Our Committee on Entertainment," Dr. Langdon Frothingham; "Usefulness of the Cow," Dr. W. E. Peterson; "The Absent Ones," Dr. J. H. Stickney; "Massachusetts Veterinary Association," Dr. J. F. Winchester; "Our Guest," Mr. D. S. J. Murphy; "Harvard Alumni," Dr. E. C. Beckett; "United States Bureau of Animal Industry," Dr. John S. Slee; "McGill Alumni," Dr. B. D. Pierce; "Watchdog of the Treasury," Dr. Henry S. Lewis.

Having done justice to all the good things served by the landlord, the evening was closed by the members standing and singing "Our Country," "Star Spangled Banner," "Should Auld Acquaintance Be Forgot."

HENRY S. LEWIS,
Secretary.

